# ERp44/CG9911 promotes fat storage in *Drosophila* adipocytes by regulating ER Ca^2+^ homeostasis

**DOI:** 10.18632/aging.203063

**Published:** 2021-05-24

**Authors:** Youkun Bi, Yan Chang, Qun Liu, Yang Mao, Kui Zhai, Yuanli Zhou, Renjie Jiao, Guangju Ji

**Affiliations:** 1 Key Laboratory of Interdisciplinary Research, Chinese Academy of Sciences, Beijing 100101, China; 2 University of Chinese Academy of Sciences, Beijing 100049, China; 3 Key Laboratory of Protein and Peptide Pharmaceutical, Institute of Biophysics, Chinese Academy of Sciences, Beijing 100101, China; 4 Sino-French Hoffmann Institute, School of Basic Medical Science, Guangzhou Medical University, Guangzhou 510182, China

**Keywords:** CG9911, lipolysis, ER homeostasis, Ca2+ signaling, ER stress

## Abstract

Fat storage is one of the important strategies employed in regulating energy homeostasis. Impaired lipid storage causes metabolic disorders in both mammals and *Drosophila*. In this study, we report CG9911, the *Drosophila* homolog of ERp44 (endoplasmic reticulum protein 44) plays a role in regulating adipose tissue fat storage. Using the CRISPR/Cas9 system, we generated a CG9911 mutant line deleting 5 bp of the coding sequence. The mutant flies exhibit phenotypes of lower bodyweight, fewer lipid droplets, reduced TAG level and increased expression of lipolysis related genes. The increased lipolysis phenotype is enhanced in the presence of ER stresses and suppressed by a reduction of the ER Ca^2+^. Moreover, loss of *CG9911 per se* results in a decrease of ER Ca^2+^ in the fat body. Together, our results reveal a novel function of CG9911 in promoting fat storage via regulating ER Ca^2+^ signal in *Drosophila*.

## INTRODUCTION

Fat is stored in the form of lipid droplets (LDs) formation of which is regulated by a variety of lipases on the LD surface [1]. The dynamics of lipolysis and lipogenesis are critical for maintaining a balanced lipid metabolism [2]. Upon nutrition restriction lipases are recruited to lipid droplets so that fatty acids will be released from triacylglycerol [3]. In mammals, adipose triglyceride lipase (ATGL) and hormone-sensitive lipase (HSL) are two key lipases that function in the process of lipolysis, respectively [4]. Obesity and lipodystrophy are two major metabolic disorders resultant from an overload or a lack of lipid storage in adipocytes [5].

*CG9911* is a predicted *Drosophila* homolog of *ERp44* which was first identified as a member of the protein disulfide isomerase (PDI) family residing in the ER of mammalian cells [6]. The crystal structure of ERp44 indicates that it has three thioredoxin domains (a,b,b’) and a flexible carboxy-terminal ER retrieval signal [7]. Recent findings show that ERp44 plays key roles in quality control of secretory proteins, ER redox regulation and cellular Ca^2+^ homeostasis [8]. As a pH-regulated chaperone, ERp44 performs the retrieval of unpolymerized subunits by forming mixed disulfides with its clients such as adiponectin [9], IgM [10], serotonin transporters [11], interleukin-12 [12] and FGE/Sumf1 [13]. ER-resident enzymes which lack the KDEL motif on the C terminal such as Prx4 [14] and Ero1 [15] oxidases are correctly localized when a covalent interaction with ERp44 occurs. At the tissue level, the absence of ERp44 leads to cardiac developmental defects [16] and hypotension [17]. Furthermore, ERp44 inhibits inositol 1,4,5 trisphosphate receptor type 1 (IP_3_R1) dependent Ca^2+^ release from ER to cytoplasm by specifically binding to the third luminal loop (L3V) of IP_3_R1 [18].

Ca^2+^ is a key signal which plays roles in a variety of cellular processes such as fertility, cell proliferation and apoptosis [19]. Endoplasmic reticulum (ER) is an important Ca^2+^ storage place where the cellular Ca^2+^ homeostasis is maintained via transmembrane Ca^2+^ channels such as the inositol 1,4,5-trisphosphate receptors (IP_3_Rs), the ryanodine receptor (RyR), the sarco/endoplasmic reticulum Ca^2+^-ATPase (SERCA) and the STIM1 (stromal interaction molecule1) [20]. Ca^2+^ signaling is also an important regulator involved in multiple adipocytic activities including adipogenesis and lipid storage. For example, *Drosophila* IP_3_R mutants are obese and hyperphagic [21]; STIM1 negatively regulates 3T3-L1 pre-adipocytes differentiation [22]; and adipogenesis is defective in human adipocytes in the absence of SERCA activity [23].

Here, we identify CG99AA/ERp44 as an important regulator of lipid storage in *Drosophila* adipocytes. *CG9911* knockout causes elevated lipolysis which is suppressed by RNAi of ER Ca2^+^ channels. Ca^2+^ imaging shows that ER Ca^2+^ store is decreased in *CG9911* mutant flies. ER stress is induced by the mutation of *CG9911* and *CG9911* mutant flies exhibit aggravated lipolysis in the fat body. We propose that decreased ER Ca^2+^ store induces ER stress which is responsible for the lipolysis alteration in the fat tissues.

## RESULTS

### Generation and characterization of the *CG9911* mutants

**Figure 1:**
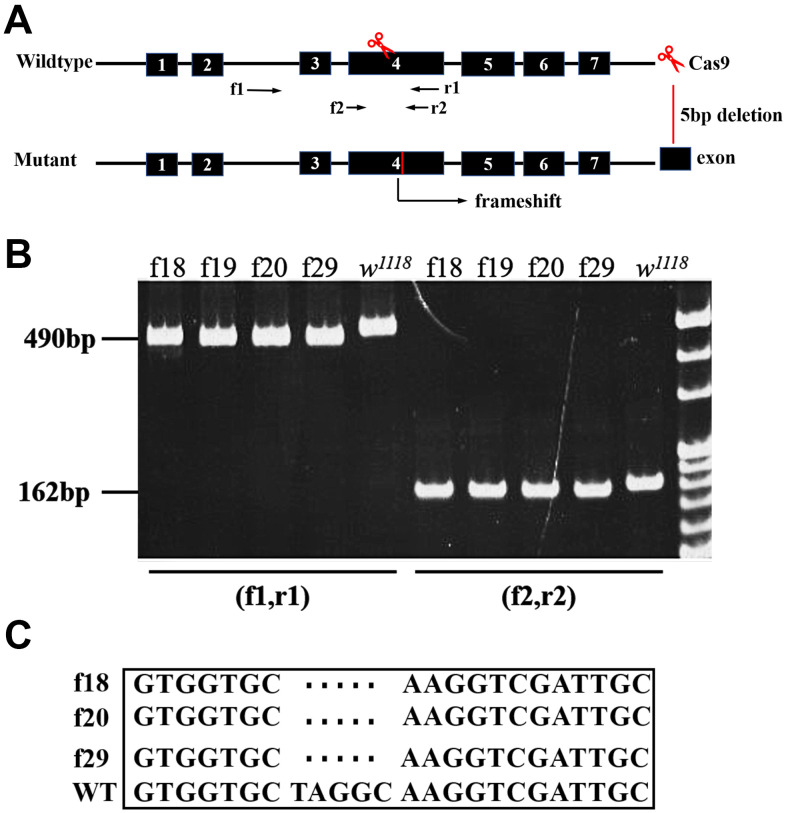
**Characterization of *CG9911* mutants.** (**A**) Schematic presentation of *CG9911* mutant mediated by CRISPR/Cas9. The scissors indicate where the CRISPR/Cas9 cleaves at the *CG9911* locus. Red line indicates 5 bp deletion on genomic region. Two pairs of identification primers are marked (f1, r1) and (f2, r2) and located by opposite arrows. (**B**) The extracted genomic DNA of homozygous lines (f18, f19, f20, f29) is used as single fly PCR templates. The different molecular weight compared to *w^1118^* indicates successful deletion. (**C**) Sequencing results show that 5 bp DNA are deleted before ‘NGG’.

The putative protein sequence of CG9911 was aligned with the orthologs from different species, including humans, using the Clustal X software (Supplementary Figure 1A), revealing a 51% sequence identity of CG9911 with the human ERp44. A highly conserved THD domain in *CG9911* with the N-terminal signal peptide, KDEL motif and an ER resident signal, on the C-terminal are depicted in Supplementary Figure 1A. Immunocytochemistry shows that CG9911 is co-localized with BiP, an ER marker, in S2 cells, indicating a potential ER-related function for CG9911 (Supplementary Figure 1B, 1C). To investigate the biological function of CG9911, mutant flies were generated using the CRISPR/Cas9 system. We used a combination of two gRNAs both targeting on the fourth exon of *CG9911* to inject *Drosophila* embryos (Figure 1A). A mutant allele, *CG9911^f20^*, which deletes 5 bp DNA of the coding sequence was obtained, and used throughout this study (Figure 1C). *CG9911^f20^* is predicted to be a null allele of *CG9911* because the deletion causes a frame shift of the open reading frame. Western blot results confirmed that no CG9911 proteins were produced in *CG9911^f20^* mutant flies (Supplementary Figure 1D). The *CG9911^f20^* mutant flies are viable and fertile with no visible morphological phenotypes under normal conditions. The developmental process of *CG9911^f20^* is 6 h delayed as compared with the wild type at the early 3rd instar stage and 20 h delayed for the enclosing adults. These results suggest that CG9911 is dispensable for viability and fertility, but may have important functions during development or under special conditions.

With a highly specific monoclonal antibody (see supplementary information for details), we analyzed the expression pattern of CG9911 in *Drosophila.* At the embryonic stages, CG9911 was ubiquitously expressed in all cells with a cytoplasmic localization (Supplementary Figure 2A). It is enriched in the eye disc (Supplementary Figure 2B), the wing disc (Supplementary Figure 2B), the oenocytes, the fat body (Supplementary Figure 2D) and the muscles (Supplementary Figure 2E) at the larval stages. It also appears in the testis and the ovary of the adult flies (Supplementary Figure 2C). Semi-quantitative expression of CG9911 was also examined with western blotting (Supplementary Figure 2F), which shows that CG9911 is expressed in almost all developmental stages with different levels in different tissues (Supplementary Figure 2G). Taken together, the results suggest that CG9911 is likely an ER protein with different levels of expression in different tissues during all developmental stages. In male adult flies, more CG9911 protein is expressed than that in female flies, which suggests CG9911 might have different roles specific for sex related functions.

### Loss of *CG9911* leads to increased lipolysis in *Drosophila* adipocytes

**Figure 2:**
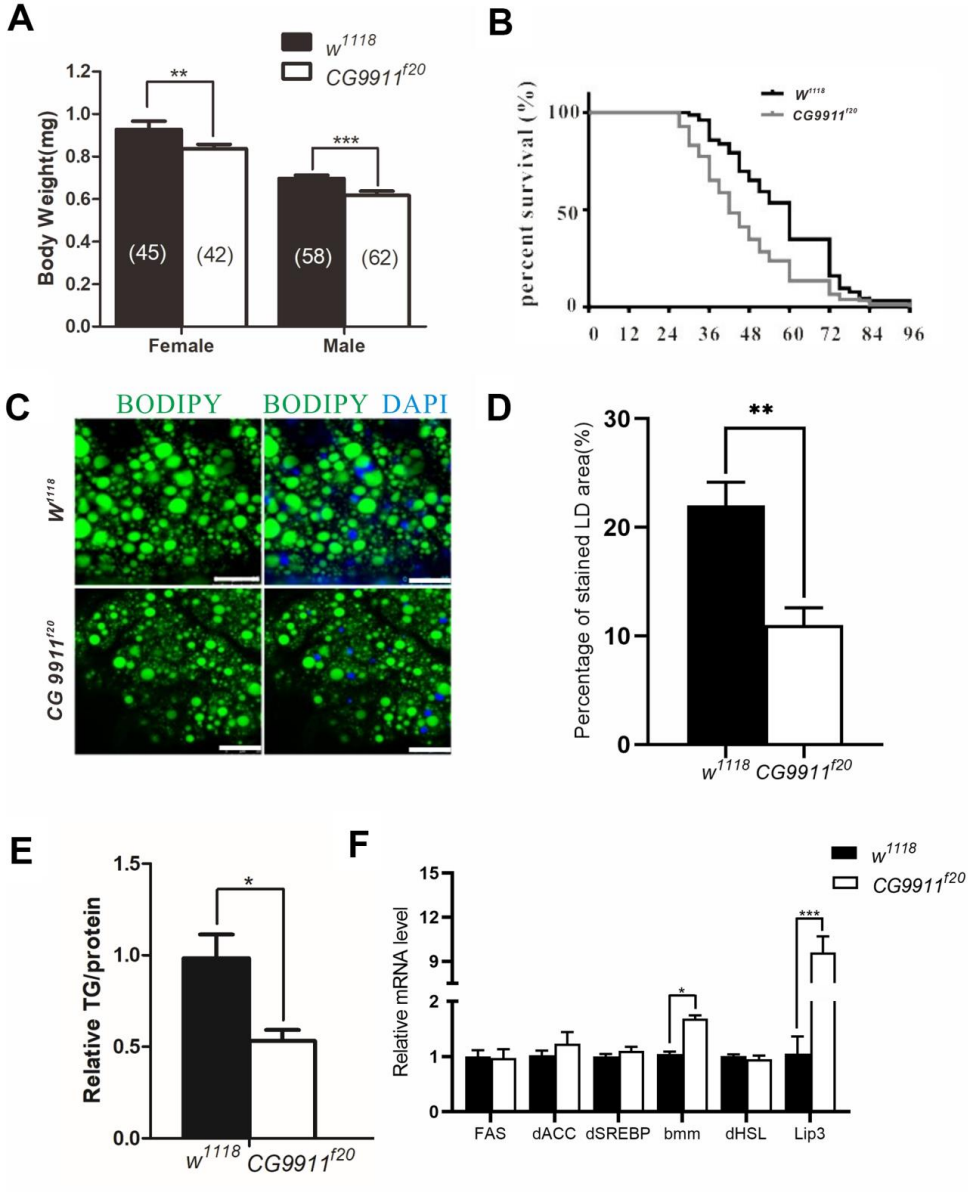
**Phenotypes of CG9911 mutant flies.** (**A**) Body weight of adult flies (1 day after eclosion) is examined in both *CG9911* mutants and *w^1118^*. The numbers of flies are indicated in bracket. (**B**) Survival curves of *CG9911* mutant and *w^1118^* flies under starvation stimulation. Log-rank (Mantel-Cox) test, p < 0.001. (**C**) LD staining in *CG9911* mutant and *w^1118^* fat body. BODIPY (green)was used to stain LD. Nuclei were stained by DAPI. Scale bar represents 25 μm. (**D**) The proportion of stained LD area in the micrograph. Over 15 micrographs in every group were considered for statistics. Data are presented as the means ± s.e.m; ** p<0.01. (**E**) Relative glyceride levels in whole body of *CG9911* mutant and *w^1118^* flies. Three independent replicates were performed. Glyceride levels were normalized to protein content; * p < 0.05. (**F**) Real-time PCR of lipogenesis and lipolysis related genes of whole body. All gene detections were subject to three independent replicates. Data are presented as the means ± s.e.m; * p < 0.05, *** p<0.001.

The bodyweight of both male and female adult flies of*CG9911^f20^* is significantly reduced compared to that of the wild type flies (Figure 2A), but the viability of the mutants remains unchanged (data not shown). However, the *CG9911* mutants exhibit significantly lower survival rate than the wild type flies under starvation conditions (Figure 2B). Given that starvation affects cellular processes such as amino acid biosynthesis, protein and lipid metabolism, glucose metabolism and lifespan determination [24], we hypothesized that *CG9911* may play a role in cellular metabolism. To test this hypothesis, we performed assays to assess lipid droplets (LDs) in the fat body (*Drosophila* fat tissue) at different developmental stages. At the larval stage, no significant differences of LD size or TAG levels were detected between the *CG9911* mutant and *w^1118^* wild type animals (Supplementary Figure 2A). Consistently, no difference on ectopic accumulation of fat was observed in *Drosophila* larval oenocytes (hepatocyte-like cells) under fed condition. *CG9911* mutant did not alter the lipid mobilization compared with wild type under starvation (Supplementary Figure 3D). Though the LD size of *CG9911* mutant was larger than *w^1118^* at selfsame development stage (Supplementary Figure 3A), TAG detection showed no difference between mutant and wild type (Supplementary Figure 3B, 3C). Interestingly, BODIPY staining shows that the abdominal LDs of the 5-day old adult flies of *CG9911^f20^* are** fewer and smaller than that of the wild type flies (Figure 2C, 2D). More strikingly, the TAG level in the whole body of *CG9911^f20^* is decreased by 46.6 % compared with that in the wild type control (Figure 2E). To verify the possible reasons for the fat decrease in the absence of *CG9911*, the expression of lipogenesis related genes (*FAS, dACC, dSREBP*) and lipolysis related genes (*bmm, dHSL, Lip3*) of whole body were examined by real time PCR (Figure 2F). Fatty acid synthase (FAS) and acetyl-CoA carboxylase (ACC) are two enzymes critical for energy production and storage in both *Drosophila* and mammals [25]. *Drosophila* sterol regulatory element-binding proteins (dSREBP) are transcription factors which regulate the synthesis of enzymes involved in sterol biosynthesis [26]. As shown in Figure 2E, expression of these lipogenesis related genes is not affected by the *CG9911* mutation. However, as the key enzymes catalyzing the hydrolysis [27], lipase3 (*Lip3*) and adipose triglyceride lipase (*bmm*) show increased expression by different folds. The expression of *bmm* in *CG9911^f20^* is ~1.6 folds of that in wild type animals, and *Lip3* is ~9 folds. These results indicate that *CG9911* loss of function results in an increase of lipolysis in the fat body of adult flies. Lipolysis is a process in which triacylglycerol is hydrolyzed into glycerol and free fatty acids (FFAs) in adipocytes and affects energy homeostasis [28]. Lipolysis can be stimulated by various factors including catecholamines, thyronines, glucocorticoids [29], TNF-α and lipopolysaccharides [30]. Lipolysis can also be increased by ER stress which is observed in the adipose tissue of burned patients and cultured human adipocytes [31]. Thus, we hypothesize that CG9911, an ER resident protein, affects lipolysis in response to ER stress.

### *CG9911* mutant cells display ER stress

**Figure 3:**
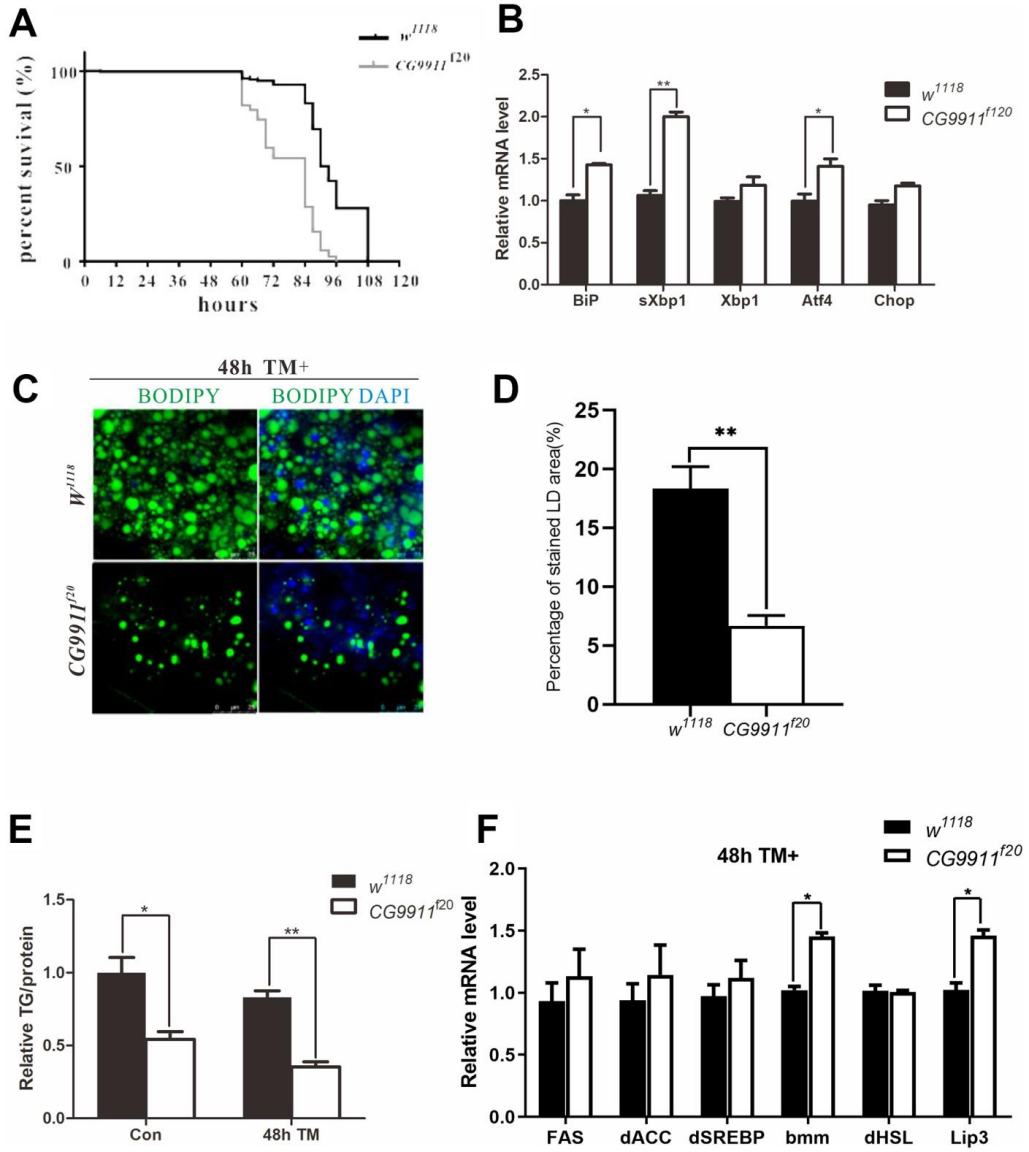
**CG9911 mutant causes ER stress.** (**A**) Survival curves of *w^1118^* and *CG9911^f20^* treated with tunicamycin. Log-rank (Mantel-Cox) test indicates p < 0.0001. (**B**) Real-time PCR detecting mRNA level of BiP, sXbp1 and Xbp1 in *w^1118^* and *CG9911^f20^* flies. Relative mRNA level was normalized to *w^1118^*. All gene detection was subject to three independent replicates. Data are presented as the means ± s.e.m; *p<0.05, ** p<0.01. (**C**) Lipid droplets staining of fat bodies in adult flies with and without TM treated. BODIPY was used to stain lipid droplets (green), and nuclei is stained by DAPI. (**D**) The proportion of stained LD area in the micrograph. Data are presented as the means ± s.e.m; ** p<0.01. (**E**) TAG level of adult flies treated with and without TM in three independent trials. Data were presented as the means ± s.e.m. * p < 0.05 and **p < 0.01. (**F**) Three independent real-time PCR detection of lipogenesis and lipolysis related genes in TM-induced flies at 48h. Data are presented as the means ± s.e.m; * p < 0.05.

ER stress occurs when ER is unable to cope with excessive unfolded or misfolded proteins accumulated in the lumen [32]. Unfolded protein response (UPR) is initiated by the activation of three transmembrane proteins of activating transcription factor 6 (ATF6), inositol requiring enzyme 1 (IRE1) and PKR-like endoplasmic reticulum kinase (PERK). At the normal conditions, ATF6, IRE1 and PERK are blocked by binding immunoglobulin protein (Bip) which is a molecular chaperone located in the lumen of ER and a target of the ER stress response [33]. To examine whether the mutation of *CG9911* is associated with ER stress, tunicamycin (TM) was used to induce ER stress in the adult flies. The survival curves show that *CG9911^f20^* is more sensitive to TM-induced ER stress as compared to the wild type (Figure 3A). In the absence of any chemical induction, *CG9911* mutants already exhibit ER stress as judged by the Bip, the spliced Xbp1 (sXbp1), and Atf4 expression, three ER stress markers (Figure 3B). Especially, the sXbp1, acting as a transcription factor, is a product of cleavage from unspliced Xbp1 by the active IRE1 [34]. The TAG level in *CG9911* mutants is decreased by 47.9% in the absence of TM induction and by 55.2% in the presence of TM induction, respectively, as compared to that of the wild type control (Figure 3E). Both the size and the number of lipid droplets are reduced in *CG9911^f20^* mutant, the phenotype of which is enhanced by TM induction (Figure 3C, 3D). Also, we investigated the fat metabolism-related genes in MT-induced files at 48h via real time PCR (Figure 3F). A notable increase in bmm and Lip3 could be observed in mutants compared with *w^1118^*. These results suggest that the lipolysis phenotype of the *CG9911^f20^* mutant is caused by increased ER stress in flies.

ER stress can be induced by various causes most of which are related with impaired folding functions of proteins resulting from genetic mutations or drug treatment [35]. In addition, Ca^2+^ level of ER is also a factor controlling ER stress in both mammals and *Drosophila*. Ca^2+^ is essential for the activity of ER resident proteins such as Calnexin and Calreticulum which are involved in the folding of glycosylated proteins [36]. Therefore, next, it is possible that Ca^2+^ level is altered by loss of *CG9911* and the phenotypes of CG9911 mutant are associated with the Ca^2+^ alteration.

### *CG9911* mutation decreases ER Ca^2+^

**Figure 4:**
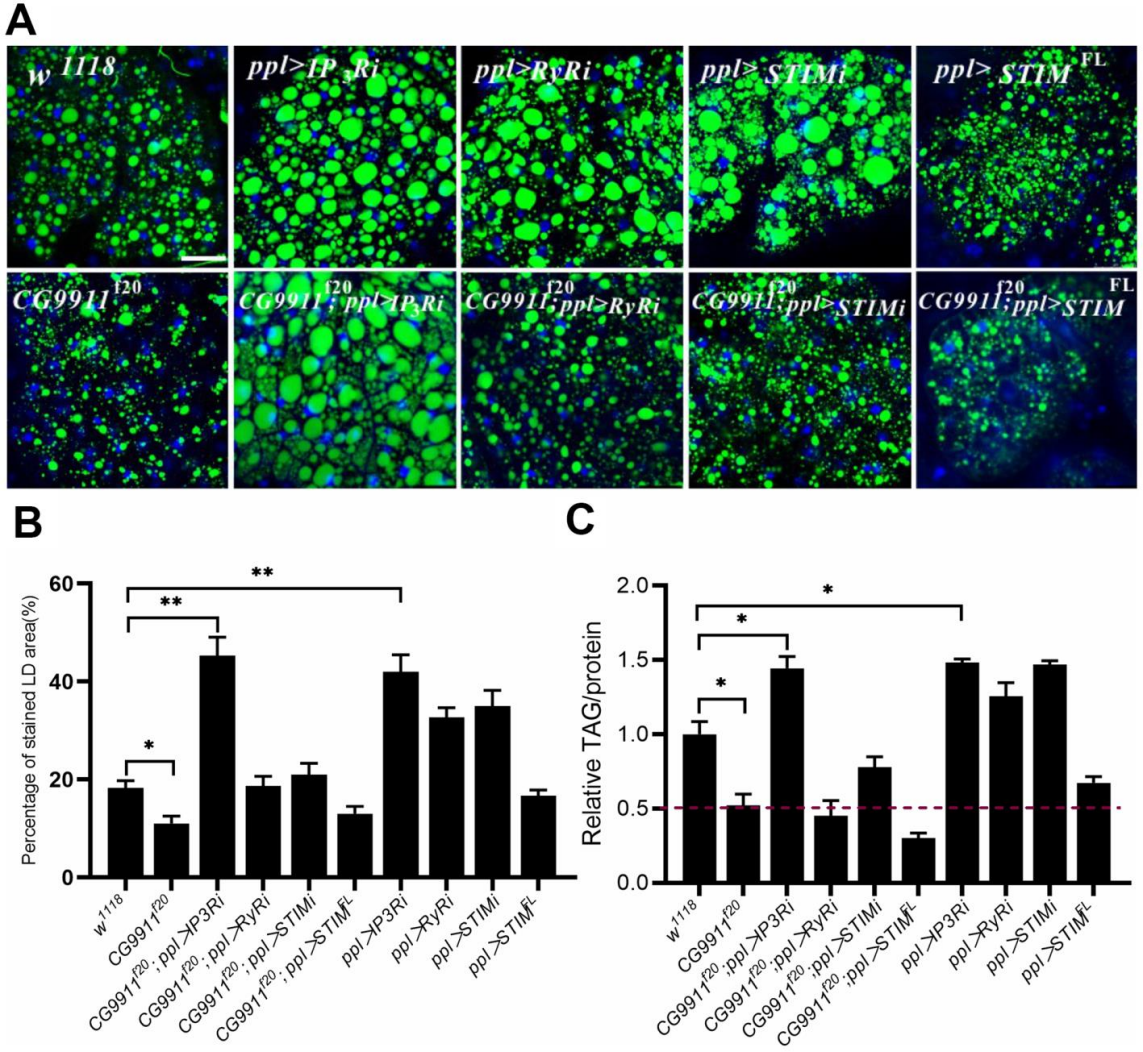
**The phenotype of CG9911 mutants is rescued by ER Ca2+ reduction.** (**A**) Knockdown of IP_3_R rescued lipid storage phenotype of *CG9911* mutants. Lipid droplets were stained by BODIPY and nuclei were stained by DAPI. Scale bar =25 μm. (**B**) The percentage of stained LD area in the micrograph. Over 10 micrographs in each group were considered for data analysis. Data are presented as the means ± s.e.m; *p<0.05, ** p<0.01. (**C**) Relative glyceride levels were examined in adult fat bodies with different genotypes in three independent trials. Glyceride levels were normalized to protein content. Data are presented as the means ± s.e.m; *p < 0.05.

It has been reported that disruption Ca^2+^ signaling leads to activation of ER stress coping response, such as UPR and mobilization of pathways to regain ER homeostasis [37, 38]. Thus we wondered whether the elevated ER stress in *CG9911* mutants is caused by Ca^2+^ flow from the ER to the cytoplasm [39]. We specifically knocked down or overexpressed *IP_3_R, RyR* and *STIM* in the fat body of *CG9911* mutants. IP_3_R and RyR are two main types of Ca^2+^ channels that regulate Ca^2+^ release from ER to the cytoplasm [20]. STIM is also a regulator of ER Ca^2+^ store through store operated Ca^2+^ entry (SOCE) [40]. *IP_3_R* depletion leads to a phenotype of obesity and increased fat storage [21]. Our results show that knockdown of *IP_3_R* in fat body promotes both the size of LDs (Figure 4A, 4B) and TAG level (Figure 4B). *IP_3_R* knockdown efficiently rescues the decrease of lipid droplets and glyceride level caused by *CG9911* deletion. *IP_3_R, RyR* and *STIM* knockdown lead to the increase of lipid droplets size (Figure 4A, 4B) and elevated glyceride content (Figure 4C). *STIM* knockdown partially alleviate the lipolysis phenotype of *CG9911* mutants (Figure 4B, 4C). Interestingly, we specially investigated the mRNA level of UPR-related genes, and results indicated that *IP3R* knockdown could reduce the UPR caused by *CG9911* deletion, which is proved by the significant decrease of *Bip*, *sXbp1*, *Xbp1*, and *Atf4* (Supplementary Figure 4). These results indicate that blocking Ca^2+^ release from ER to cytoplasm by knockdown of *IP_3_R* significantly suppresses the phenotype of lipolysis in *CG9911* mutant, implying that ER Ca^2+^ signal has contributions to the lipolysis phenotype caused by depletion of *CG9911*.

### CG9911 is required for ER Ca^2+^ homeostasis

**Figure 5:**
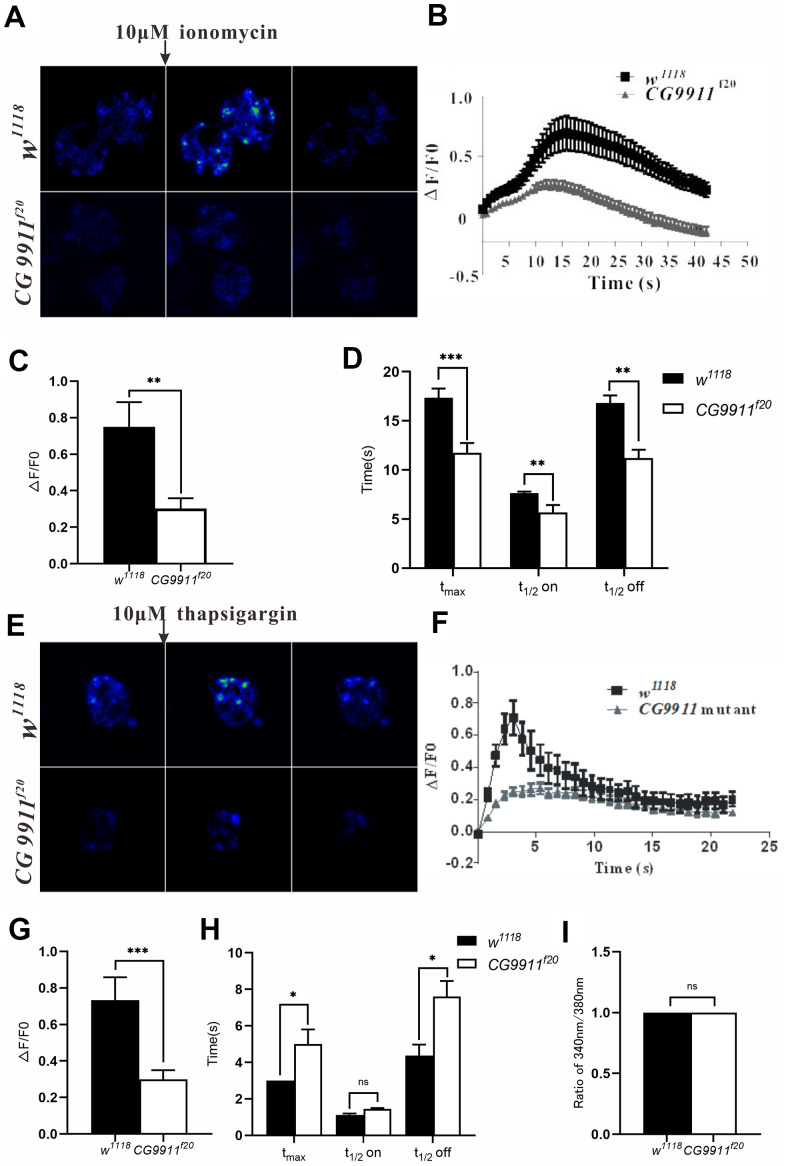
**CG9911 modulates intracellular calcium homeostasis.** (**A**) Fluorescence changes of *w^1118^* and *CG9911* mutant fat bodies which were treated with 10μM inomycin. Scale bar = 50 μm. (**B**) The dynamic changes of fluorescence intensity(ΔF/F_0_) in the signal fat cell of *w^1118^* and *CG9911* mutant files when stimulated with 10μM inomycin. (**C**) The difference of mean F/F_0_ between *w^1118^* and *CG9911* mutant fat cells when treated with 10μM inomycin. Data are presented as the means ± s.e.m; ** p<0.01. (**D**) The comparation of the mean data for time from basal to peak (t_max_), half time raise (t_1/2_ on), and decay (t_1/2_ off) between *w^1118^* and *CG9911* mutant fat cells when treated with 10μM inomycin. Data are presented as the means ± s.e.m; ** p<0.01, *** p<0.001. (**E**) Ca^2+^ imaging of *w^1118^* and *CG9911* mutant fat bodies when treated with 10 μM TG. (**F**) The dynamic changes of fluorescence intensity(ΔF/F_0_) in the signal fat cell of *w^1118^* and *CG9911* mutant files when stimulated with 10μM TG. (**G**) The difference of mean data of F/F_0_ between *w^1118^* and *CG9911* mutant fat bodies when treated with 10 μM TG. Data are presented as the means ± s.e.m; *** p<0.001. (**H**) The comparation of the mean data for time from basal to peak (t_max_), half time raise (t_1/2_ on), and decay (t_1/2_ off) between *w^1118^* and *CG9911* mutant fat cells when treated with 10μM TG. Data are presented as the means ± s.e.m; *p<0.05. (**I**) The quiescent Ca^2+^ level in the signal fat cell of *w^1118^* and *CG9911* mutant files.

Given the fact that the increased lipolysis phenotype of *CG9911* mutant is suppressed by the down-regulation of Ca^2+^ channel, we reasoned that CG9911 may play a role in ER Ca^2+^ homeostasis. To test this hypothesis, Ca^2+^ imaging experiments were performed with the fat body of *CG9911* mutant and the wild type files. Cells with Fluo-4 AM, Ca^2+^ indicator, were treated with 10 μM ionomycin (an ionophore that binds Ca^2+^) to stimulate Ca^2+^ release from ER to cytosol (Figure 5A). The fluorescence signals were recorded to calculate the ER calcium level (Figure 5B). Compared to the wild type, the amplitude of ionomycin induced Ca^2+^ transients in *CG9911* mutants is decreased by 57% (Figure 5C). Dynamically, Ca^2+^ release in *CG9911* mutant fat body is different from that in wild type animals: the time of fluorescence signal from the basal level to the peak (t_max_), the half time of rise (t_1/2_on) and half time of decay (t_1/2_ off) are shortened by 32.6%, 29.1% and 37.1%, respectively (Figure 5D). In accordance with the results by ionomycin treatment, Ca^2+^ release induced by 10 μM thapsigargin (TG, the non-competitive inhibitor of SERCA) is reduced by 58.4% (Figure 5E, 5G) with t_max_ and t_1/2_ off increased by 68.7% and 83.7% (Figure 5H). Previous study revealed that ERp44 affects Ca^2+^ homeostasis by regulating the activity of IP_3_R1 in mammalian cells [41]. To check whether CG9911 affects cytosol Ca^2+^ level, we performed the experiments of the relative resting Ca^2+^infat body of *CG9911^f20^* flies and the wild type flies. Individual fat body cells of adult flies were incubated with Fura-2 AM. Our results show that the resting Ca^2+^ level presented by the fluorescence ratio of 340 nm/380 nm is not significantly different between *CG9911* mutant and the wild type (Figure 5I). Together, these results indicate that CG9911 plays a role in maintaining ER Ca^2+^ homeostasis in *Drosophila* fat body. The resting Ca^2+^ signaling is unlikely contributes to the increased lipolysis phenotype of *CG9911* mutant flies.

## DISCUSSION

**Figure 6:**
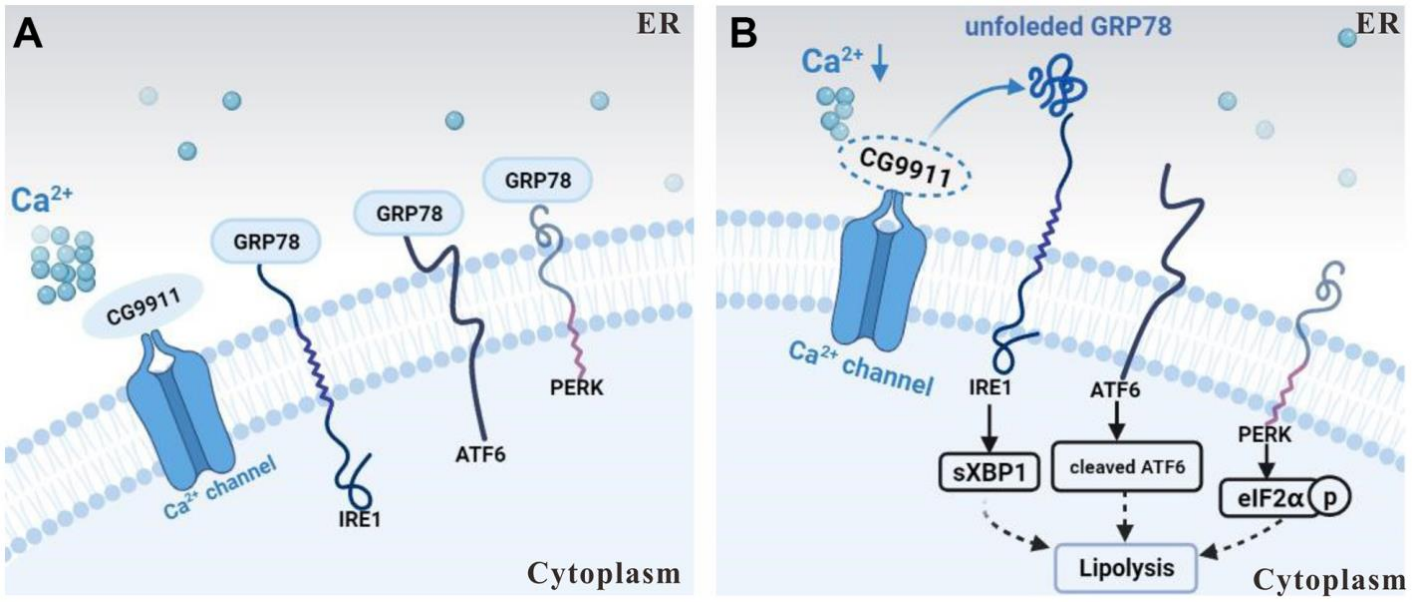
**Schematic of CG9911 function in lipolysis.** (**A**) *CG9911* was expressed in wild type flies. Under normal condition, with *CG9911* expression, ER stress effectors are inhibited by GRP78. (**B**) *CG9911* knockout induced lipolysis in fat cells. Without *CG9911* expression (dashed circle), ER stress effectors are active and ER Ca^2+^ level (a cluster of small circles) is decreased. Lowercase p in a circle means phosphorylation.

In this study, we show that loss of *CG9911* causes a reduction of ER Ca^2+^ and induces ER stress in fat cells. The induced ER stress then triggers unfolded protein response (UPR) as indicated by the activation of three effectors (IRE1, ATF6 and PERK) [42], which leads to the expression of lipolysis related genes in *Drosophila* adipocytes (Figure 6A, 6B). Our results indicate that CG9911 promotes adipose tissue fat storage by regulating ER Ca^2+^ homeostasis and CG9911 likely functions as an inhibitor of Ca^2+^ flux from the ER to the cytoplasm to maintain Ca^2+^ homeostasis in *Drosophila* adipocytes. Impaired CG9911 activity leads to reduced ER Ca^2+^, promoting lipolysis in *Drosophila* fat body.

By using the CRISPR/Cas9 system, we generated *CG9911* mutants whose phenotypes include: 1) *CG9911* mutant flies exhibit lower bodyweight and more sensitivity to starvation; 2) both the size and number of lipid droplets are decreased and the mutant animals exhibit lower TAG level than the wild type; 3) The mRNA level of lipolysis related genes, *bmm* and *Lip3*, is elevated in adult flies. Some of the phenotypes except for the body weight are observed only in the fat body of adult flies. Apart from the fat body, oenocytes are the tissue where lipolysis occurs as well. However, our data indicated that *CG9911* played roles only in the adult fat body.

Previously we reported that ERp44 (CG9911 homolog) modulates intracellular Ca^2+^ signaling by interaction with IP_3_R1 in mammalian cells [41]. Specially, ERp44 directly binds to the third luminal loop of IP3R1 depending on pH, Ca^2+^ concentration, and redox state, further to inhibit the Ca^2+^ overload [18, 43]. As the homolog of ERp44, CG9911 may has similar function in regulating the Ca^2+^ signal in *Drosophila*. Consistently, in our present study, we find that phenotypes of *CG9911* mutants are fully rescued by knockdown of *IP_3_R* in fat body in *Drosophila*. This finding suggests that *CG9911* has a genetic interaction with *IP_3_R* and thus potentially possible to regulate Ca^2+^ homeostasis by physical interaction with *IP_3_R*.

Ca^2+^ imaging experiments show that *CG9911* loss of function leads to down regulation of ER Ca^2+^ without affecting cytosol resting Ca^2+^ level in adult fat bodies. Without *CG9911*, decreased ER Ca^2+^ acts as a trigger to ER stress. ER stress has been connected to transcriptional activation in the regulation of lipid metabolism [44]. ER stress causes the increase of lipid storage both in mammalian hepatic [45] and fat cells though different ways. According to our data, CG9911 only affects lipid storage in fat bodies but not in oenocytes, which implies that CG9911 has a tissue-specific manner in lipid metabolism. The interaction of ER stress and ER Ca^2+^ homeostasis has been discussed for years due to their collective regulation of transcription and protein maturation [46]. Although modulation of ER Ca^2+^ homeostasis by CG9911 is important, other ways through which CG9911 affects ER stress cannot be ruled out. A few questions remain to be explored. For example, lipolysis is largely regulated by the intracellular concentration of cyclic adenosine monophosphate (cAMP) and by the activation of cAMP-dependent protein kinase A (PKA) in mammalian cells [47]. In our present study, whether ER stress induced by CG9911 depletion through cAMP/PKA pathway is unknown.

Our findings may contribute to understand the interactions of Ca^2+^ signaling and lipid metabolism. It may also be a hint to study the function of ERp44 and the mechanism of relevant metabolic diseases. Our study also sheds new light on explaining the interaction between Ca^2+^ signaling and lipid metabolism.

## MATERIALS AND METHODS

### Fly stocks

All flies were kept on standard medium containing cornmeal, yeast agar, soybean, syrup and molasses [48]. The culture condition is 25° C and 60% humidity unless otherwise stated. *ppl-GAL4* (#105013) and *Oe-GAL4* (#113874) stocks were obtained from *Drosophila* Genomics Resource Center (DGRC). Other stocks used were from the Bloomington Stock Center.

### Generation of CG9911 mutants

By CRISPR/Cas9 system, we selected two target DNA binding site on the third exon according to the rules [49]. We followed the target sequence design principle: 5’GG-N17–19-NGG3’. The sequences of two gRNAs utilized in this study are 5’-GGATACTTGTTTATGTGGAAACGG-3’ and 5’- GGAAAAGTGGTGCTAGGCAAGG-3’. After transcribed *in vitro* as previously described [50], the mRNAs and gRNAs were mixed to a final concentration of 800 ng/μl for the mRNA, 862 ng/μl and 613 ng/μl for the gRNAs, respectively. The mixture was centrifuged at maximum speed for 10 min before microinjection to *w^1118^* embyos. Inheritable F0 which yielded enough F1 were collected for screen by using single fly PCR with the pair of primers: 5’-TGCAGCGTCTAATGAATTGG-3’ and 5’-GTCCCTACGATCGAAGTAGC-3’. Positive PCR fragment from homozygous flies were used for sequencing.

### Generation of CG9911 antibody

The CDS fragment of CG9911 isoform C was cloned from cDNA of *drosophila* embryos and then subcloned into pGEX-6p-1 (GE healthcare, UAS) by EcoR I and Sal I. An N-terminal, GST-fused CG9911 protein was expressed in *E.coli* BL21 (DE3) strain (Trangene, Beijing, China) induced by 40 μM IPTG at 16° C for 18 h. The protein was purified using Glutathione Sepharose 4B (GE Healthcare, UAS) according to a standard protocol. GST-tag was removed by PreScission Protease (GenScript, NJ, USA) in cleavage buffer (50 mM Tris-HCl, 150 mM NaCl, 1 mM EDTA, 1 mM DTT, pH 7.0) at 4° C overnight. The purified CG9911 protein was used as antigen at the concentration of 400 μg/ml in PBS and mixed with 20 μg/ml CpG ODN (5’-tccatgacgttcctgacgtt-3’thiophosphorylated) as adjuvant. Each of 8 week-old female BALB/C mouse was immunized with 500μl antigen by intraperitoneal (i.p.) injection. The second booster injection was 30 days after the primary immunization dose. The third, fourth and fifth booster injection went 15, 7 and 3 days after the last injection, respectively. After five booster injections, the spleen B lymphocytes isolated from immunized mice and SP2/0-Ag14 myeloma cells were fused in the presence of PEG4000 (Merck) at 37° C overnight. The hybridoma cells were cultured in complete IMDM medium (GIBCO, USA) supplemented with 20% FBS (GIBCO, USA), 1% penicillin/streptomycin (GIBCO, USA) and HT (GIBCO, USA). After 15 days feeding in 96-well plates, the hybridomas were screened by using ELISA. To gain monoclones, the cells from positive wells were recloned by limiting dilution and identified under an inverted microscope as previously described (Yokoyama et al, 2006). The positive monoclonal hybridoma cells tested by ELISA again were expended to 24-well plates and incubated for 1 week at 37° C, 5% CO_2_. Monoclonal antibodies were purified from collected cell supernatant by using protein G column (GEHealthcare, USA).

### ELISA assay

Polystyrene 96-well plates were coated with250μg purified CG9911 protein in 50μl PBS at 4° C overnight and blocked with block buffer (0.5% BSA, 0.05% Tween-20 in PBS) for 1 h at room temperature. After 3 times washing with PBST (0.05% Tween-20 in PBS), the plates were loaded with 100μl supernatant of hybridoma cells for 1 h. Subsequently,100μlgoat anti-mouse Ig-HRP (1:10000 diluted in block buffer) was added to each well and incubated for 1h.After 3 washes, the plate was developed by using 100μl TMB substrate (eBioscience, USA) per well for 5 min. The reaction was stopped by adding50 μl 2N H_2_SO_4_and liquid color was changing from blue to yellow. The absorbance was measured at 450 nm using a Spectrophotometer (Thermo Fisher, Finland) and Multiskan GO v1.00.40.

### Western blot

Flies of different developmental period were homogenized in cold RIPA lysis buffer (50mMTris, 150mMNaCl, 1% Triton X-100, 1% sodium deoxycholate, 0.1% SDS, pH 7.4) in the presence of 1 mM PMSF and protease inhibitor cocktail (Cell Signaling Technology, USA) and centrifuged at 12000 rpm, 4° C for 20 min to remove insoluble precipitates. The concentration of total protein was determined by Bradford (sigma, USA) and was boiled in 2×SDS loading buffer for 15 min. The detected samples have the same total protein concentration and were separated by SDS-PAGE, transferred onto PVDF membranes (Millipore), and incubated with primary antibodies against CG9911 (1:1000, Cell Signaling Technology), β-actin (1:1000, Proteintech). The membranes were incubated with the appropriate HRP-conjugated secondary antibodies (1:10 000, Abways), and the signals were analyzed using the SuperLumina ECL HRP Substrate Kit (Abbkine, USA). The position of the target protein is referenced to the color prestained marker (Cofitt, HongKong). A minimum of three independent analysis were performed, and typical example is presented.

### Immunohistochemistry

Wandering third instar larvae and adult flies of *w^1118^* were collected and dissected in cold PBS. Wing discs, fat body, oenocytes, muscles, ovary, testis and other tissues were separately fixed in 4% paraformaldehyde in PBS for 40 minutes at room temperature followed with 3 washes of PBST (0.3% Triton X-100 in PBS). After blocked in 10% goat serum in PBST for 1 h, tissues were incubated with CG9911 antibody (1:100) at 4° C overnight. Subsequently, fluorescent secondary antibody was used for signal detection. For Lipid droplet staining in adult abdominal fat bodies, 5 days adult flies of the correct genotypes were collected and dissected as previously described. After 3 washed in PBS, fat bodies were incubated in PBS with 1 mg/ml BODIPY 493/503 (Invitrogen, USA) and 2 ng/ml DAPI (Beyotime Biotech, China) for 40 min at room temperature. The embryos of *w^1118^* were stained with CG9911 antibody according to standard protocol. All fluorescent images were captured by a Leica confocal microscope SP5 (Leica, Germany).

### Triglycerides measurements

Whole adult flies glyceride quantification was determined as described. 10 adult flies were collected (three groups per genotype) and homogenized in PBST (0.5% Tween in PBS). The supernatants were measured with TAG reagent (Sigma, UAS).

### Calcium imaging in fly fat cells

Fat body cells from*w^1118^*and CG9911 mutant flies (1 day after eclosion) were dissected in hemolymph-like (HL) buffer (128 mM NaCl, 2 mM KCl, 35.5 mM sucrose, 4 mM MgCl_2_, 1.8 mM CaCl_2_, 5 mM HEPES, pH 7.4) as previously described. Individual cells were loaded with 5 mMFluo-4 AM (Invitrogen, UAS) on poly-L-Lysine (sigma, UAS) coated wells at 37° C for 30 min in the dark. After perfused with calcium-free HL buffer (128 mM NaCl, 2 mM KCl, 35.5 mM sucrose, 4 mM MgCl_2_, 2 mM EDTA, 5 mM HEPES, pH 7.4), cells were stimulated by 10 μM ionomycin (Beyotime Biotech, China) or 10 μM thapsigargin (Sigma, USA) to cause Ca^2+^ release from ER. The fluorescence signal was recorded by using a SP5 confocal microscope (Leica, Germany) connected to an inverted microscope (Leica, Germany). The Ca^2+^dependent fluorescence intensity ratio (ΔF/F_0_) was used to present the Ca^2+^release signal in 30 fat cells. The resting Ca^2+^ measurement were performed as previously described. Fat cells were incubated with 10μM Fura-2 AM (Invitrogen, UAS) in HL buffer at 37° C for 30 min. Photometric measurements were performed by using cellˆR system (Olympus, Japan) and operated at an excitation wavelength of 340 and 380 nm. The relative resting Ca^2+^ signal was presented by a ratio of 340/380 nm by using Olympus cellˆR Software.

### Quantitative real time PCR

Total RNA was extracted from adult flies by using TRIzol reagent (Invitrogen, USA). 2μg of RNA was reverse transcribed to cDNA by using PrimeScript™ RT reagent Kit with gDNA Eraser (TAKARA, Japan). Quantitative real-time PCR was performed using EvaGreen 2× qPCR Mix (abm, Canada) on a Roter-Gene machine (QIAGEN, Germany) following the manufacturer’s instructions. *β-actin* and *rp49* were used as endogenous control. See Supplementary Table 1 for q-PCR primer sequences.

### Starvation assay

50 flies of *w^1118^*and *CG9911* mutant (3 days after elcosion) were kept in vials only with PBS at normal condition. The number of dead flies was recorded every 3 hours with 3 independent groups employed. Data was analyzed by Log-rank (Mantel-Cox) test.

### ER stress assay

To induce ER stress, 100 files with different genotypes were transferred to vials containing 1% agar, 5% sucrose and 12 μM tunicamycin (Sangon Biotech, China) as previously reported. The flies were kept in normal conditions and counted every 3 hours.

### Statistical analysis

Statistical analysis was performed using GraphPad. Data were tested for significance using the Student’s test and shown as mean ± SEM. Data from three groups were compared by one-way ANOVA.

## Supplementary Material

Supplementary Figures

Supplementary Table 1
