# Supplementary material for: ERp44/CG9911 promotes fat storage in Drosophila adipocytes by regulating ER Ca2+ homeostasis
Source: Aging (Albany NY). 2021 May 24;13(11):15013–31. doi: 10.18632/aging.203063 (PMC8221293; doi:10.18632/aging.203063)
Supplement: Supplementary Figures [file aging-13-203063-s001.pdf]

SUPPLEMENTARY FIGURES

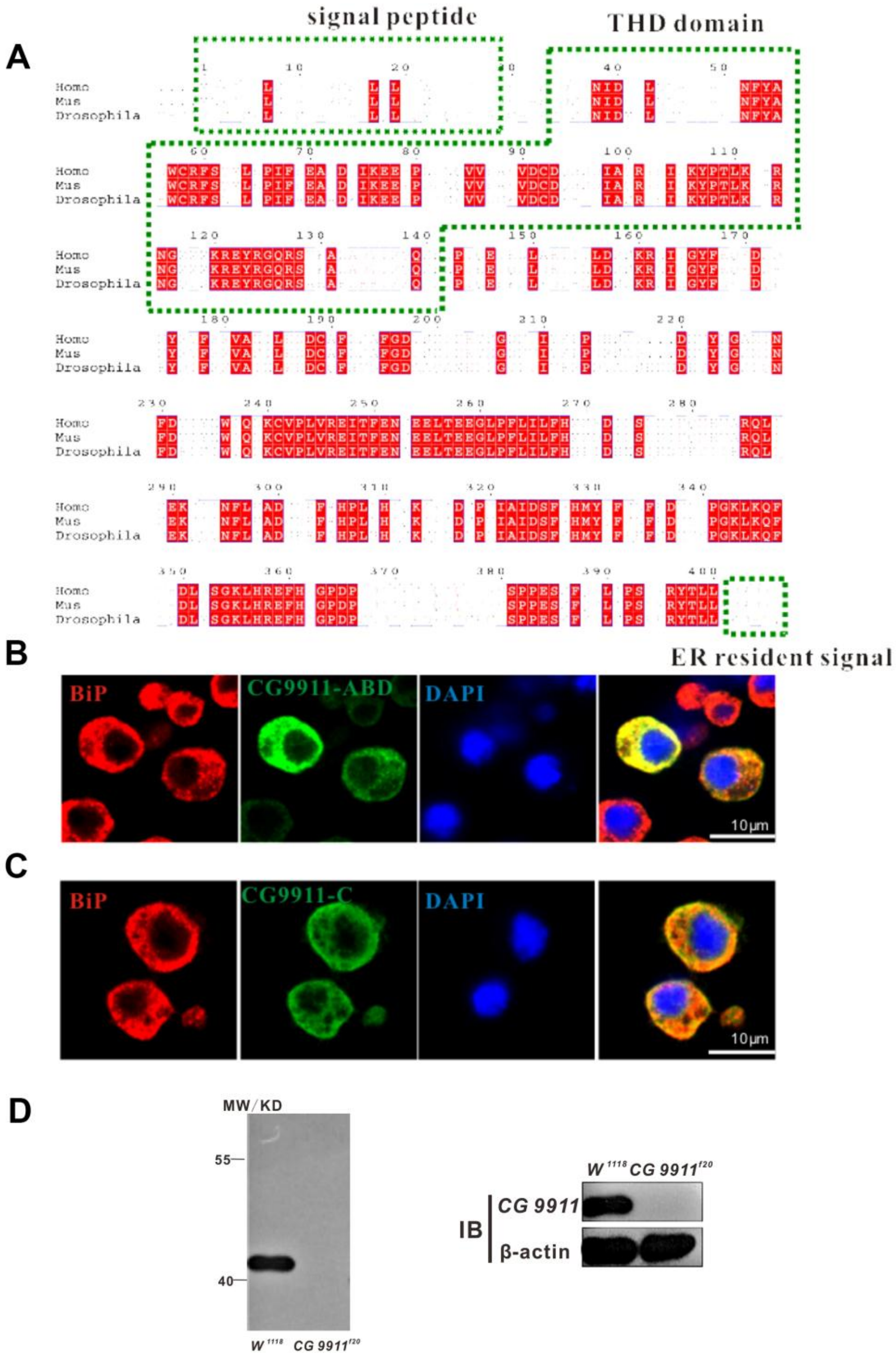

Supplementary Figure 1. CG9911 is a conserved protein. (**A**) Blast result of protein sequence in human, mouse and *Drosophila*. Identical protein sequence is presented in orange box and positive protein sequences are in transparent box. Signal peptide, THD domain and ER resident signal are marked by green dashed lines. (**B**, **C**) Subcellular location of CG9911 in S2 cells. BiP is labeled with red fluorescence signal. *CG9911* ABD isoform (**B**) and C isoform (**C**) are labeled with green fluorescence signal. DAPI is used to stain nuclei. Scale bar = 10 µm. (**D**) CG9911 protein is absent in mutant flies as shown by Western blot. Protein extracts from w^{1118} (wild type) and *CG9911* mutant flies are blotted with anti-CG9911 antibodies. β-Actin is used as loading control.

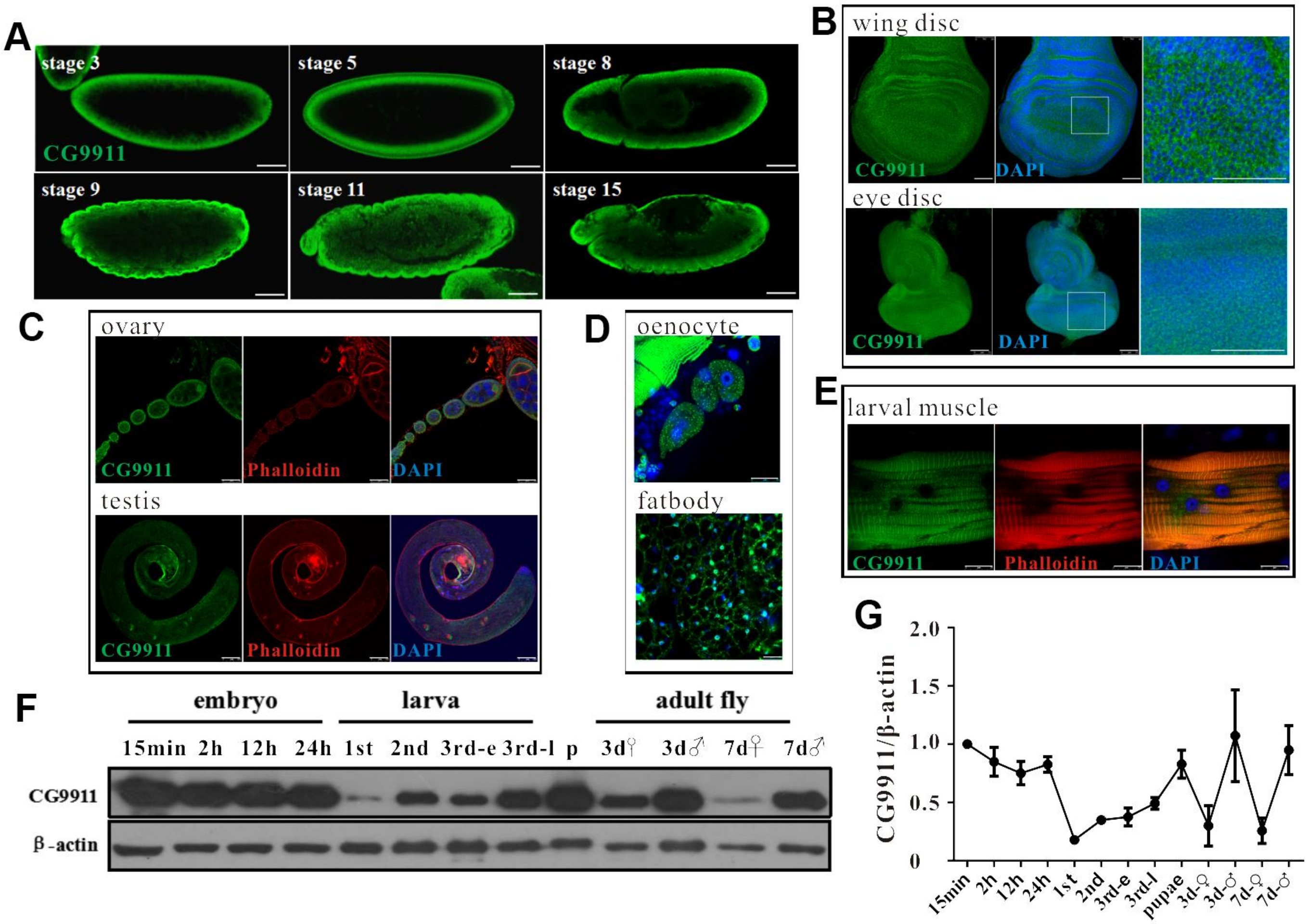

Supplementary Figure 2. Expression pattern of CG9911. (**A**) CG9911 is expressed in embryos and marked with green fluoresce signal. Scale bar = 75 µm. (**B**) CG9911 is expressed in larval eye and wing disc with enlarged view. Green signal represents CG9911, nuclei is stained by DAPI. Scale bar = 50 µm. (**C**) CG9911 expression in ovary and testis. F-actin is stained by Phalloidin. Scale car = 50 µm. (**D**) in larval oenocyte and fat body in adult fly. Scale bar = 25 µm. (**E**) in larval muscle. Scale bar = 25 µm. (**F**) western blot of flies in different developmental stages with CG9911 antibody. β-actin is used as loading control. 3rd e and 3rd l stands for early and late third instar larvae. P stands for pupae. 3d means adult flies with 3 days after eclosion. (**G**) Quantity of CG9911 expression.

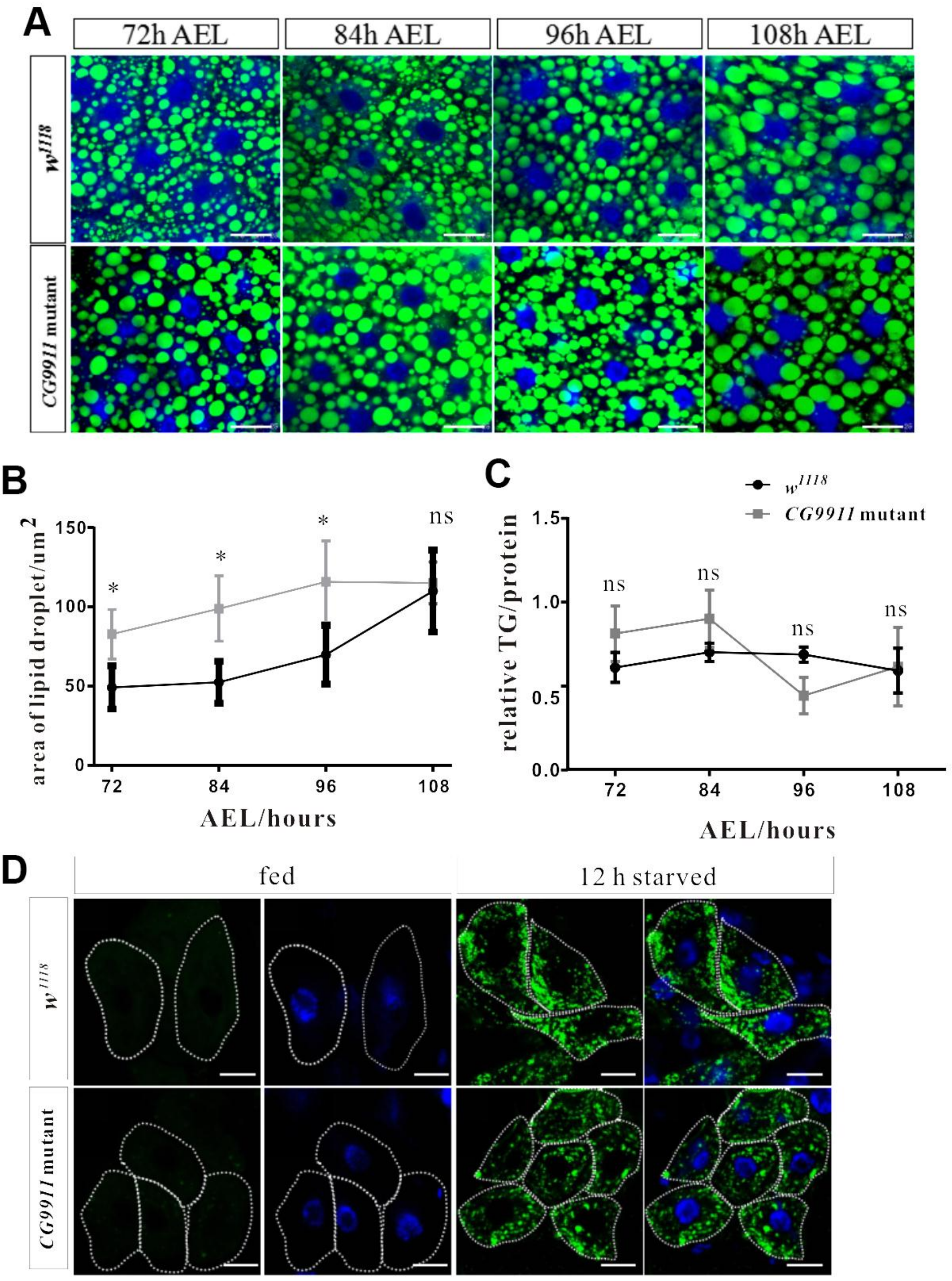

Supplementary Figure 3. CG9911 does not affect lipid metabolism of larval fat body and oenocyte. (**A**) lipid droplets staining of larval fat body in different developmental stages. Lipid droplets are stained by BODIPY and nuclei is stained by DAPI. Scale bar = 25 µm. (**B**) statistical analysis of LD size of different stages in CG9911 mutant and wild type larvae. (**C**) Relative TG level of different stages in CG9911 mutant and wild type larvae. (**D**) LD staining of oenocyte under the condition of normal fed and starvation. Lipid droplets are stained by BODIPY and nuclei is stained by DAPI. Scalebar =25 µm. Data are presented as the means ± s.e.m. *p < 0.05.

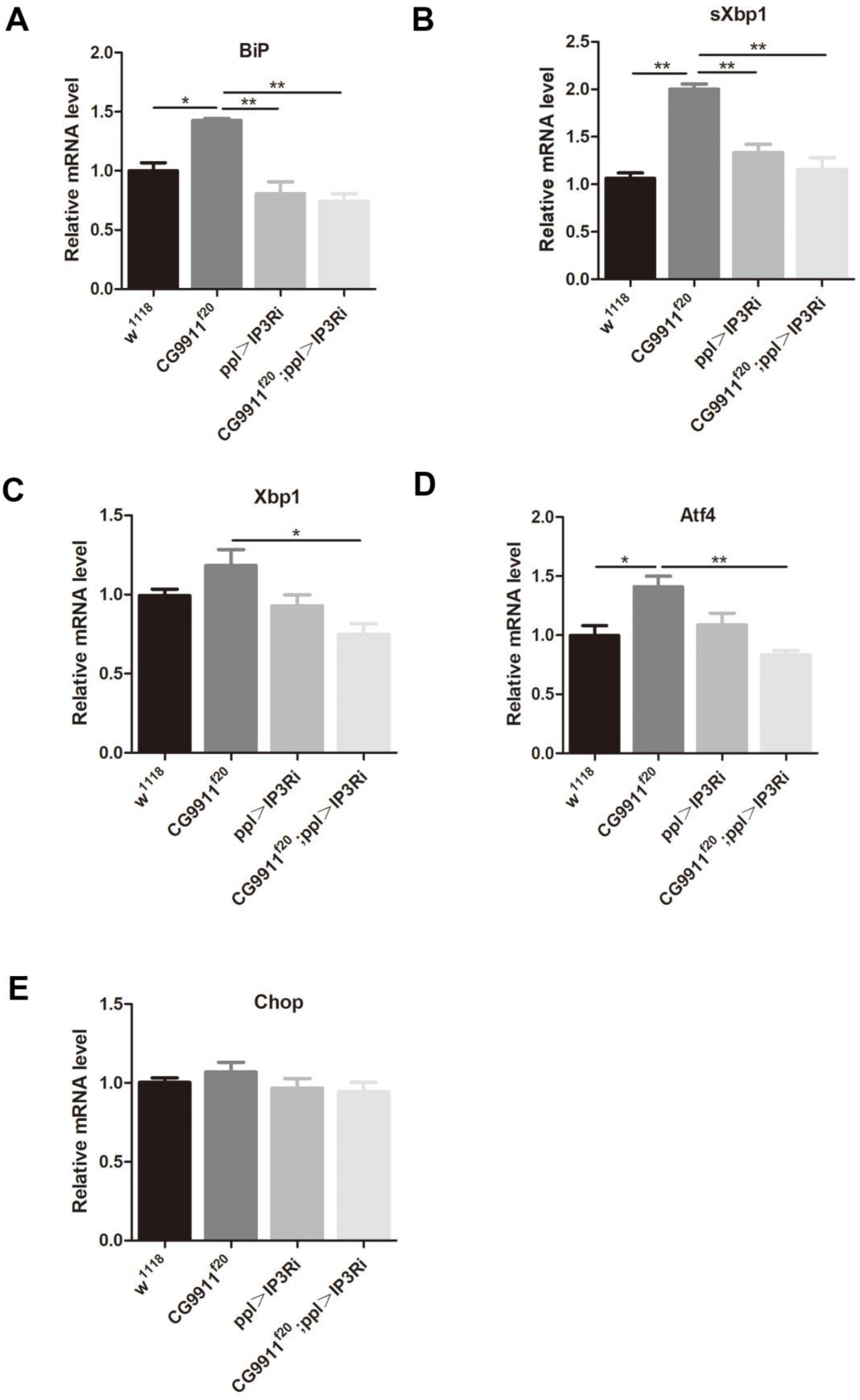

Supplementary Figure 4. Real-time PCR detection of UPR marker genes in *w^{118}*, *$CG9911^{f20}$*, *ppl>IP3Ri*, and *CG9911; ppl>IP3Ri* flies, respectably. BiP (**A**), sXbp1 (**B**), Xbp1 (**C**), Atf4 (**D**), and Chop (**E**) were subjected to investigation, respectively. Three independent repeat tests were performed in each group. Data are presented as the means ± s.e.m; * $p < 0.05$, ** $p<0.01$.
