# Supplementary material for: ERp44/CG9911 promotes fat storage in Drosophila adipocytes by regulating ER Ca2+ homeostasis
Source: Aging (Albany NY). 2021 May 24;13(11):15013–31. doi: 10.18632/aging.203063 (PMC8221293; doi:10.18632/aging.203063)
Supplement: Supplementary Table 1 [file aging-13-203063-s002.pdf]

SUPPLEMENTARY TABLE

Supplementary Table 1. Q-PCR primer sequences in this study.

Target gene	Forward primer (5'→3')	Reverse primer (5'→3')
β-actin	CTCGTACGTGGGTGATGAGG	ACATACATGGCGGGTGTGTT
rp49	ACGTTGTGCACCAGGAACTT	ACGTTGTGCACCAGGAACTT
BiP	CTGGTGTTATTGCCGGTCTG	CTGGTGTTATTGCCGGTCTG
sXbp1	CAACCTTGGATCTGCCGCAG	GACTTTCGGCCAGCTCTTCG
dSREBP	GCATTATGATGGCACTATTCG	AACGTAGCTCCTGCGTTTG
dACC	TACGATGTAGAGTCGCAGTTC	TACGATGTAGAGTCGCAGTTC
dFAS	GTGCGTCCTATCAGCTACCC	GTGCGTCCTATCAGCTACCC
Lip3	CGGGTGAATCTTCCAACCGA	GCATTGCCCATCCACACATC
dHSL	CGAGTGCCAGATGGTCTGTT	CGAGTGCCAGATGGTCTGTT
bmm	CGAGTGCCAGATGGTCTGTT	CGAGTGCCAGATGGTCTGTT
